# Development of a UPLC-MS/MS Method for the Quantification of VS-5584 and Its Application in Pharmacokinetic Studies in Rats

**DOI:** 10.1155/2020/8811522

**Published:** 2020-12-16

**Authors:** Chunling Zhou, Xiaoxin Li, Aiping He, Tianzhu Liu, Jinmiao Tian, Meihua Jiang, Lina Fang

**Affiliations:** ^1^Liaoning Institute for Drug Control, Liaoning Inspection, Examination & Certification Centre, Shenyang 110035, China; ^2^College of Pharmacy, Shenyang Medical College, Shenyang 110034, China

## Abstract

VS-5584 is a small-molecular compound that showed equivalent activity against mTOR and all class I PI3K isoforms and demonstrated preclinical activity in diverse cancer cell lines and xenograft tumor model, and rational combination of VS-5584 and other target therapies achieved promising outcomes in oncology. In the present study, we established and validated a simple and sensitive UPLC-MS/MS method for the determination of VS-5584 in plasma samples. VS-5584 was separated via an Acquity UPLC BEH C18 column, with a mobile phase composed of acetonitrile and 0.2% formic acid in water (40 : 60). The calibration curve displayed a good linearity in the range of 1.0–1000 ng/mL, with satisfactory accuracy (−13.6% < RE% < 8.8%) and precision (CV%, less than 9.2%). The validated method was then applied to a pharmacokinetic study in rats. After administration of 10 mg/kg, VS-5584 was absorbed quickly and reached a peak concentration of 473.2 ± 72.0 ng/mL after 20 min. The established method allows for the quantification of VS-5584 in rat plasma in detail and can be utilized to successfully describe the pharmacokinetic profile of VS-5584.

## 1. Introduction

The increased activity of the phosphoinositide 3-kinase (PI3K)/mammalian target of rapamycin (mTOR) signalling pathway has been associated with increased growth, survival, and proliferation in many human cancers [1]. Thus, the PI3K/mTOR signalling pathway represents an important therapeutic target to induce cancer cell death [2]. The inhibition of PI3K/AKT/mTOR pathway is regarded as a therapeutic approach in the treatment in oncology [3–5].

VS-5584 is a small-molecular inhibitor that shows equivalent activity against mTOR and all four class I PI3K isoforms [6]. It can permeate cells, including PC3, NCl-N87, HuH7, Colo205, and MDA-MB-231, to modulate signalling pathways downstream of the PI3K/mTOR pathways [7]. VS-5584 was shown to inhibit the survival and proliferation of melanoma cells (A-2058, A375, and SK-MEL-3), induce caspase-dependent apoptotic cell death *in vitro*, and further suppress A375 melanoma xenograft growth in nude mice after oral administration [8]. Treatment with VS-5584 inhibited human osteosarcoma cell (U2OS and MG-63) growth by inducing G1-phase arrest via the inhibition of PI3K/mTOR and MAPK signalling pathways to downregulate HIF-1*α* and VEGF expression [9]. Another research showed that VS-5584 was highly cytotoxic in multiple myeloma cell lines and triggered apoptosis in patient cells with a favorable therapeutic index [10]. Besides the anticancer activity, VS-5584 was also reported to suppress thrombus formation *in vitro* and *in vivo* [11].

In addition, the rational combination of VS-5584 and other targeted therapies have shown promising outcomes [12–15]. In a preclinical xenograft model of pancreatic ductal adenocarcinoma, the combination therapy with an ERK inhibitor SCH772984 increased the tumor suppression rate to 80% compared with VS-5584 treatment alone (28%) [14]. Furthermore, due to the preferential targeting of cancer stem cells (CSCs) [16, 17], VS-5584 suppressed cisplatin-resistant CSC growth and delayed tumor regrowth after cessation of cisplatin treatment in xenograft models of small cell lung cancer [16].

Although numerous findings support the clinical development of VS-5584, no detailed analytical method of VS-5584 detection has been reported, and no pharmacokinetic study has been performed. As LC-MS/MS is widely used for the detection of small molecules in biological matrices [18, 19], we established and validated a simple and sensitive ultraperformance liquid chromatography-mass spectrometry (UPLC-MS/MS) method for the quantification of VS-5584 levels in rat plasma. The pharmacokinetic profile after oral administration of VS-5584 in adult male Sprague Dawley (SD) rats was also investigated.

## 2. Materials and Methods

### 2.1. Materials and Reagents

**Figure 1:**
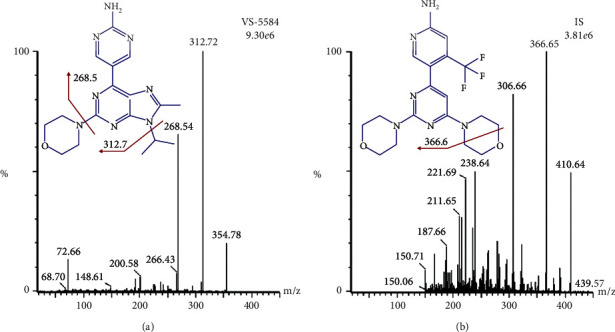
Chemical structures, precursor, and product spectra. (a) VS-5584 and (b) Buparlisib, IS.

Adult male SD rats were provided by Liaoning Changsheng Biotechnology Co., Ltd. (Benxi, China). VS-5584 (>98.3%) and buparlisib (>98.5%, internal standard, IS) were provided by AZBIOCHEM Biotechnology Co., Ltd. (Shanghai, China). HPLC-grade acetonitrile, methanol, and formic acid were obtained from Fisher Scientific (Fair Lawn, NJ, USA). The purified water for UPLC analysis was obtained from Wahaha Co. Ltd. (Hangzhou, China). The chemical structures of VS-5584 and buparlisib are displayed in Figure 1.

### 2.2. Instruments and UPLC-MS/MS Conditions

The Acquity UPLC system (Waters, USA), equipped with a UPLC binary solvent manager, an autosampler and column oven, was employed for the chromatography analysis. VS-5584 and IS were separated from interfering endogenous compounds via an Acquity UPLC BEH C_18_ (2.1 × 100 mm, 1.7 *μ*m particle diameter, Waters, USA) column under 25°C, with a mobile phase composed of acetonitrile and 0.2% formic acid in water (40 : 60) with a flow rate of 0.20 mL/min.

The mass spectrometric detection of the analytes was performed on a Waters TQ-S mass spectrometer (Waters, USA). MS-optimized parameters were listed as follows: nitrogen was used as the desolvation gas with a flow of 800 L/Hr, the desolvation temperature was set at 500°C, and capillary voltage was 2.5 kV. The MRM transitions of *m/z* 354.78 ⟶ 312.73 for VS-5584 and 410.64 ⟶ 366.65 for the IS were monitored under positive electrospray ion (ESI^+^) condition. The optimized cone voltage of VS-5584 and IS was 40 V, and the collision energy for VS-5584 and IS was 24 V and 32 V, respectively. Dwell time for both of the analytes was 0.025 s.

### 2.3. Stock Solutions, Calibration Standards, and Quality Control Samples

Stock solutions of VS-5584 and IS were dissolved in methanol to obtain a concentration of 1.0 mg/mL, respectively. The stock solution of VS-5584 was further diluted in methanol to generate a working solution in the concentration range of 5.0–5000 ng/mL. Then, 10 *μ*L of corresponding working solutions was added to 50 *μ*L of drug-free rat plasma to generate calibration standards of VS-5584 (1.0, 2.5, 10, 50, 250, 500, and 1000 ng/mL). Quality control (QC) samples were prepared at LLOQ, low, medium, and high concentrations (1.0, 2.0, 200, and 800 ng/mL) separately in the same manner. The IS working solution was prepared in acetonitrile at a concentration of 40 ng/mL.

### 2.4. Sample Treatment

Plasma samples were purified by protein precipitation with acetonitrile. Briefly, 50 *μ*L of rat plasma was added with 300 *μ*L of IS working solution. This mixture was vortexed for 1 min before centrifugation at 900 × g for 5 min at 4°C. After filtration, 1 *μ*L of the supernatant was introduced to the UPLC-MS/MS instrument for analysis.

### 2.5. Method Validation

All validation procedures were performed following the US FDA guidelines.

Selectivity was assessed by comparing blank plasma samples from six individual rats with the spiked samples at the lower limit of quantitation (LLOQ).

Calibration curves were obtained by plotting the peak area ratio of VS-5584 and IS against VS-5584 concentration. A 1/*X*^2^ weighted least-square linear regression method was utilized, where ‘*X*' represented the concentration of VS-5584. The calibrators should be less than ±15% of nominal concentrations, except at the LLOQ where the calibrator should be ±20%.

The impact of carryover on the accuracy of the sample concentration was assessed by injecting blank samples after the upper limit of quantitation (ULOQ). The carryover should not exceed 20% of LLOQ.

QC samples at LLOQ, low, medium, and high concentrations in six replicates were assessed over three independent runs to evaluate accuracy and precision. For low, medium, and high QC samples, accuracy (relative error (RE%)) and precision (coefficient of variation (CV%)) should be less than ±15%, while the acceptance criteria were increased to ±20% for LLOQ.

Extraction recovery and matrix effect of VS-5584 were assessed in six different samples at four QC levels. Recovery was calculated by the ratio of the peak area of the extracted sample to that of blanks spiked with VS-5584 postextraction. The matrix effect was expressed by the ratio of the blanks spiked with VS-5584 postextraction to that of the corresponding amount of VS-5584 prepared in acetonitrile: water (300 : 50, *v/v*).

Stability studies were conducted under a variety of different conditions. The accuracy and precision under the above conditions should be within ±15%.

### 2.6. Pharmacokinetic Application

SD rats of SPF grade (male, 215–235 g) were kept in a climate-controlled room. After acclimatisation for 5 days, six rats were orally treated with VS-5584 (10 mg/kg) to assess the pharmacokinetic profile. For administration, VS-5584 was first dissolved in DMSO, added with PEG-400, and further diluted with water to obtain a concentration of 1.0 mg/mL (the mixed solvent containing 5% DMSO, 20% PEG-400, and 75% water). Blood samples were collected from the suborbital vein at 5, 10, 20, 30, and 45 min and 1, 2, 4, 6, 8, 10, and 12 h after dosing. Plasma was obtained after centrifuging blood samples at 800 × g for 5 min and stored at −25°C until analysis. All pharmacokinetic parameters were calculated based on a noncompartmental model with Phoenix WinNonlin software (version 8.0).

## 3. Results and Discussion

### 3.1. Method Optimization

To get a favorable mass response, VS-5584 and IS were infused directly into the mass spectrometer with a syringe pump, respectively. Better responses were achieved in positive ionization mode as VS-5584 and IS are nitrogenous compounds. Ion *m*/*z* of 354.78 for VS-5584 and 410.64 for IS was detected as precursors [M + H]^+^, and an *m*/*z* of 312.73 for VS-5584 and 366.65 for IS were defined as product ions, as these were the most abundant product ions in the MS^2^ scan spectra, respectively. Additionally, the ionization of *m/z* 354.78 → 312.73 for VS-5584 and 410.64 → 366.65 for IS was stable and reproducible. The MRM reaction of IS was consistent with previous reports [18, 19].

To obtain suitable retention time and high detection sensitivity, the mobile phase system was determined in acetonitrile, methanol, and proportions of acid in water. As the organic phase in the mobile phase, acetonitrile achieved a smoother baseline in the IS channel. Then, 40% acetonitrile was chosen, compared to 35% and 30% acetonitrile in the mobile phase, resulting in a total run time of 3.0 min. Subsequently, a mixture of acetonitrile and water containing 0.2% formic acid (40 : 60) at a flow rate of 0.20 mL/min was chosen as the mobile phase system.

To simplify the sample treatment, plasma proteins were precipitated prior to treatment. Acetonitrile and methanol were tested as extraction solvents. Satisfactory recoveries were acquired with both of the two solvents, but acetonitrile yielded a thinner IS peak shape.

### 3.2. Method Validation

#### 3.2.1. Selectivity

**Figure 2:**
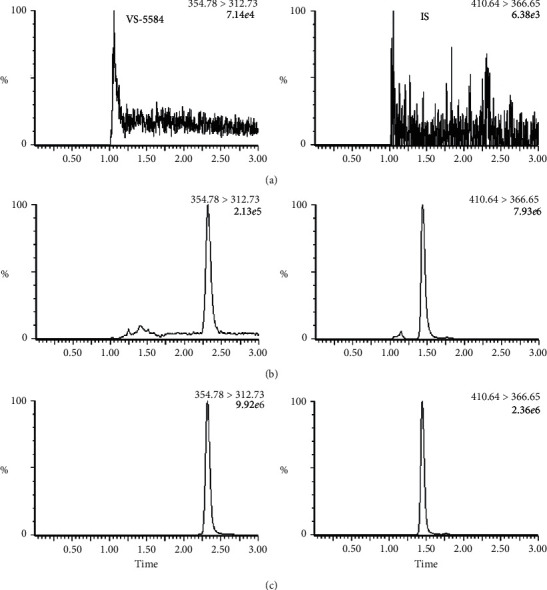
Representative chromatograms: (a) blank plasma sample; (b) blank plasma sample spiked with VS-5584 (LLOQ) and IS; and (c) rat plasma sample collected at 0.5 h after dosing.

Blank samples were free of endogenous interfering substances at the retention times of VS-5584 (2.32 min) and IS (1.45 min), indicating sufficient selectivity of this method. Representative MRM chromatograms of blank plasma, VS-5584, and the IS in rat plasma are presented in Figure 2.

#### 3.2.2. Linearity, LLOQ, and Carryover

In rat plasma, the calibration curve of VS-5584 demonstrated good linearity in the range of 1.0–1000 ng/mL (typical equation: *y* = 0.03554*x* + 0.00632, *r* = 0.9977). The LLOQ was determined to be 1.0 ng/mL.

After injection of ULOQ, no obvious chromatographic peak was detected in blank samples at the retention times of VS-5584 and the IS; thus, no carryover was found in this method.

#### 3.2.3. Accuracy and Precision

As shown in Table 1, the accuracy of VS-5584 in three independent runs ranged from −9.5% to 7.0% for low, medium, and high QCs, and within −13.6% for LLOQ. The intraday and interday precisions for VS-5584 were less than 9.2% for LLOQ, and 4.8% to 5.9% for the other QC concentration levels.

#### 3.2.4. Recovery and Matrix Effect

The percentage recoveries of VS-5584 and IS spiked in rat plasma were found to be greater than 89.3%. Table 1 shows the average matrix effects obtained for four QC levels of VS-5584. These data suggest that there was no measurable matrix effect interfering with the detection of VS-5584 in rat plasma.

#### 3.2.5. Stabilities

Table 2 summarized the stability data of VS-5584 at low, medium, and high QC levels after storage. VS-5584 was stable at −25°C for 30 days, under room temperature for 12 h and after three freeze-thaw cycles in rat plasma. The processed samples remained intact at 4°C in an autosampler for 12 h.

### 3.3. Pharmacokinetic Application

**Figure 3:**
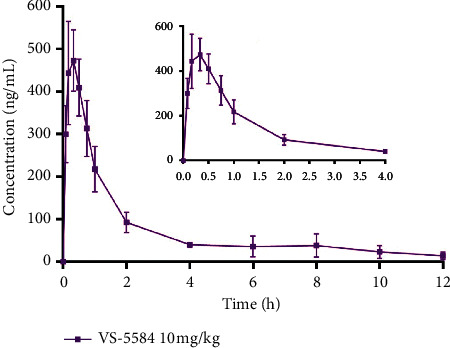
Mean plasma concentration-time profile of VS-5584 (*n* = 6).

The established method was successfully used for the first time to study the pharmacokinetic behaviour of VS-5584 in rats after an oral administration of 10 mg/kg. The time course of plasma VS-5584 concentrations is shown in Figure 3. VS-5584 was absorbed quickly and reached a peak concentration of 473.2 ± 72.0 ng/mL 20 min after drug administration. VS-5584 exhibited a biexponential disposition. Plasma concentration of VS-5584 decayed rapidly by 2.0 h after administration and eliminated slowly with a *t*_1/2_ of 3.28 ± 1.37 h at the terminal phase. AUC_0_ _⟶_ _12_ _h_, AUC_0_ _⟶_ _∞_ were found to be 865.2 ± 192.1 and 928.8 ± 228.0 h ng/mL, respectively. *V*_z_/F and CL/F were found to be 54.1 ± 29.6 L/kg and 11.3 ± 2.5 L/h/kg, respectively.

## 4. Conclusions

To our knowledge, we established and validated for the first time a simple and sensitive UPLC-MS/MS method for the determination of VS-5584 in rat plasma. The established analytical method was successfully applied to describe the pharmacokinetic profile of VS-5584 after oral administration in rats. The established method is suitable for future preclinical and clinical studies such as pharmacokinetic studies and drug-drug interaction studies. Additionally, the established method can be used as a reference for developing a high-throughput bioassay and for therapeutic monitoring of VS-5584 in human plasma.

## Figures and Tables

**Table 1:** Precision, accuracy, matrix effect, and recovery for analysis of VS-5584 in rat plasma (*n* = 6).

Spiked concentration (ng/mL)	Intraday precision CV (%)	Interday precision CV (%)	Accuracy (RE%)	Matrix effect (%, mean ± SD)	Recovery (%, mean ± SD)
1.0	9.2	8.0	−13.6	93.3 ± 6.9	92.4 ± 5.5
2.0	5.9	7.0	−9.5	105.3 ± 6.2	92.0 ± 4.4
200	4.8	7.0	8.8	94.1 ± 1.8	89.4 ± 3.9
800	5.5	5.3	8.2	98.8 ± 1.9	90.8 ± 3.9
IS	—	—	—	90.3 ± 9.8	89.3 ± 3.8

**Table 2:** Stability of VS-5584 in rat plasma (*n* = 3).

Conditions	VS-5584		
	Spiked (ng/mL)	RE (%)	CV (%)
30 days at −25°C	2.0	9.4	8.7
	200	−12.2	12.5
	800	−5.7	5.0

Three freeze–thaw cycles	2.0	−6.5	6.6
	200	−6.5	5.6
	800	7.4	5.9

12 h at room temperature	2.0	−7.0	8.2
	200	10.7	7.7
	800	9.2	5.8

4°C in autosampler for 12 h in processed samples	2.0	−8.7	5.4
	200	10.4	8.7
	800	9.9	4.8

## Data Availability

All the data related to these findings are included in the manuscript.
